# Use of Artificial Intelligence for Predicting Parameters of Sustainable Concrete and Raw Ingredient Effects and Interactions

**DOI:** 10.3390/ma15155207

**Published:** 2022-07-27

**Authors:** Muhammad Nasir Amin, Waqas Ahmad, Kaffayatullah Khan, Ayaz Ahmad, Sohaib Nazar, Anas Abdulalim Alabdullah

**Affiliations:** 1 Department of Civil and Environmental Engineering, College of Engineering, King Faisal University, Al-Ahsa 31982, Saudi Arabia; kkhan@kfu.edu.sa (K.K.); 218038024@student.kfu.edu.sa (A.A.A.); 2 Department of Civil Engineering, COMSATS University Islamabad, Abbottabad 22060, Pakistan; waqasahmad@cuiatd.edu.pk (W.A.); sohaibnazar@cuiatd.edu.pk (S.N.); 3 MaREI Centre, Ryan Institute and School of Engineering, College of Science and Engineering, National University of Ireland Galway, H91 HX31 Galway, Ireland; a.ahmad8@nuigalway.ie

**Keywords:** pollution, waste, aggregate, concrete, building, construction

## Abstract

Incorporating waste material, such as recycled coarse aggregate concrete (RCAC), into construction material can reduce environmental pollution. It is also well-known that the inferior properties of recycled aggregates (RAs), when incorporated into concrete, can impact its mechanical properties, and it is necessary to evaluate the optimal performance. Accordingly, artificial intelligence has been used recently to evaluate the performance of concrete compressive behaviour for different types of construction material. Therefore, supervised machine learning techniques, i.e., DT-XG Boost, DT-Gradient Boosting, SVM-Bagging, and SVM-Adaboost, are executed in the current study to predict RCAC’s compressive strength. Additionally, SHapley Additive exPlanations (SHAP) analysis shows the influence of input parameters on the compressive strength of RCAC and the interactions between them. The correlation coefficient (R^2^), root mean square error (RMSE), and mean absolute error (MAE) are used to assess the model’s performance. Subsequently, the k-fold cross-validation method is executed to validate the model’s performance. The R^2^ value of 0.98 from DT-Gradient Boosting supersedes those of the other methods, i.e., DT- XG Boost, SVM-Bagging, and SVM-Adaboost. The DT-Gradient Boosting model, with a higher R^2^ value and lower error (i.e., MAE, RMSE) values, had a better performance than the other ensemble techniques. The application of machine learning techniques for the prediction of concrete properties would consume fewer resources and take less time and effort for scholars in the respective engineering field. The forecasting of the proposed DT-Gradient Boosting models is in close agreement with the actual experimental results, as indicated by the assessment output showing the improved estimation of RCAC’s compressive strength.

## 1. Introduction

**Figure 1:**
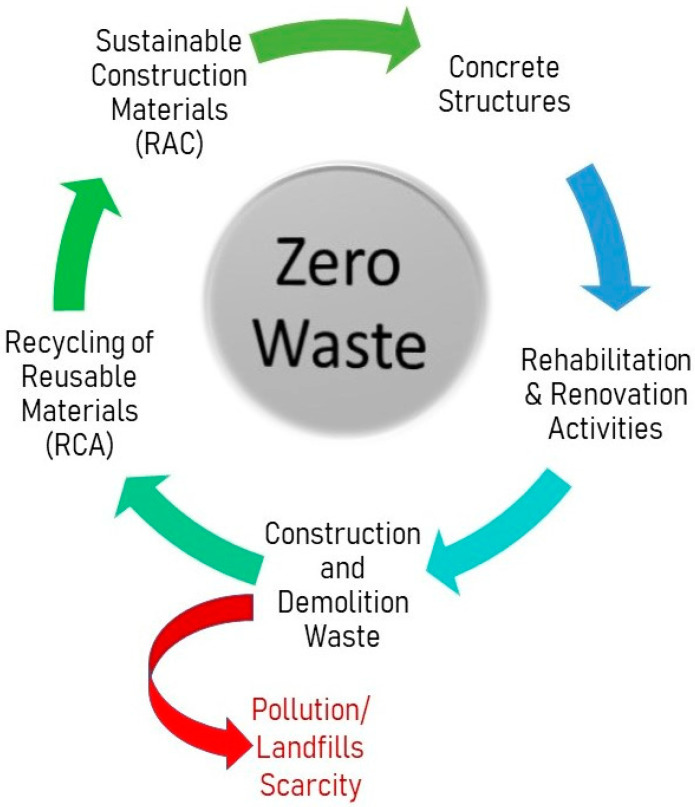
Concrete cycle—production to recycling.

The rapid growth in population and industry has enhanced the demolition rate of ancient structures in order to advance construction. In this way, a large amount of material waste is usually deposited in landfills, contributing to the depletion of landfill areas and, ultimately, environmental problems [1,2,3,4,5]. In the last two decades, recycled aggregate (RA), generated as a result of demolition waste, has been utilised as an alternative to natural aggregates in manufacturing concrete. This incorporation of RA has come out with enhanced resource sustainability in the field of construction and with reduced environmental impact [6,7,8,9]. Industrial by-products and waste fibre have already been used to partially replace cement and other concrete ingredients in previous studies [10,11,12,13,14,15,16,17,18]. Natural resources can be conserved, and the negative environmental impact of the construction industry can be reduced by using RA as coarse aggregates in new concrete mixes [19,20,21,22,23]. However, due to variation in the properties of RAs, the mechanical properties of concrete with RA also vary compared to that of conventional natural aggregate concrete (CNAC). Accordingly, it is necessary to understand the relation between recycled coarse aggregate concrete (RCAC) mix proportions and the respective mechanical properties before adopting material on a large scale in the construction industry. Using recycled aggregate concrete (RAC) instead of virgin concrete can lead to a significant reduction in cost, natural resource consumption, landfill scarcity, and environmental pollution (Figure 1).

Machine learning (ML) techniques, due to advancements in artificial intelligence (AI), are now applicable to forecasting the mechanical properties of concrete [24,25,26]. ML methods such as clustering, classification, and regression may be applied to effectively foresee various parameters and forecast the concrete ultrasonic pulse velocity with high precision. Due to recent advancements in AI, supervised ML algorithms may help to estimate the mechanical behaviours of different material types [27]. Regression clustering and classification are among the ML techniques implemented to statistically process and forecast the precise compressive strength of concrete [28]. The application of ML methods to predict high-calcium FA-GPC was also considered in a previous study. Adaptive boosting (AdaBoost), Bagging (Bagging), and Decision tree (DT) techniques have also been considered to forecast FA-GPC compressive strength. Previous research has included regression, error distribution process, sensitivity analysis, model validation, and statistical checks for comparing and validating the precision of deployed algorithms. The deployment of both individual (i.e., DT) and ensembled ML (i.e., Bagging) for the prediction of FA-GPC compressive strength is the novelty of the current study. Artificial intelligence (AI) procedures, with technological development, have provided computer-aided modelling techniques with more reliability and accuracy to address structural engineering problems [29,30,31,32]. The prediction of RCAC mechanical properties by applying computational intelligence and machine learning (ML) techniques is also gaining the attention of researchers [4]. Younis and Pilakoutas [20] presented a model to forecast RCAC compressive strength using non-linear and multi-linear regression analysis. Duan et al. [33] and Sahoo et al. [34] predicted the RCAC compressive strength by using artificial neural network (ANN) techniques. Deshpande et al. [35] used the model tree, ANN, and non-linear regression techniques to predict RCAC compressive strength. Recently, genetic programming and multivariable regression models have also been utilised by González-Taboada et al. [36] to forecast RCAC’s elastic modulus, compressive strength, and splitting tensile strength. The reduction in the consumption of effort, time, and cost for experimentation by using ML techniques in order to forecast concrete properties can profit the civil engineering field.

In addition, the post-hoc model-agnostic method named SHapley Additive exPlanations (SHAP) may also be applied to provide insight into ML models [37,38]. The structure–characteristic correlation interpretation in various fields, such as material science [37,38,39], the corrosion rate for low-alloy steel estimation [40], the behaviour of nanophotonic structures [41], the synthesis of innovative inorganic materials [42], text classification [43], finances [44], and the field of biomedical engineering [37,38,39], can effectively be achieved by employing SHAP. However, the employment of interpretable machine learning using SHAP is not familiar enough for concrete properties [45]. The integration of ML algorithms with SHAP was performed in this study to provide better insight into concrete mix design, in terms of mechanical strength via its complex and non-linear behaviour, and to describe the contribution of input factors via a linkage of every input parameter with a weight factor. Overall, via the implementation of interpretable machine learning approaches through SHAP, this study establishes a valuable structure–characteristic correlation that could be beneficial for the sustainable development of durable concrete mixes.

Various researchers have investigated the effects of RA on different properties, such as strengths under compressive and splitting tensile loadings, etc., of concrete, which multiple researchers have explored. The compressive strength of concrete is reduced when RA is incorporated instead of natural aggregates [46]. In concrete with RA, the aggregate is surrounded by mortar, leading to lower density and a higher water absorption (WA) capacity of the RA, which ultimately influences the concrete compressive strength [47]. However, the workability is also reduced in the case of RA compared to that in the case of natural aggregates. Furthermore, the poor bonding between mortar and RA is also one of the primary reasons for the decreased compressive strength of recycled concrete [48]. The concrete properties are reduced by up to 10–25% when replacing natural aggregates with RAs, depicting the considerably reduced compressive strength of concrete in the case of 100% substitution [49,50]. Therefore, to avoid the consumption of testing time, cost, and raw materials, prediction models based on experimental data are usually developed to forecast the concrete compressive strength [4,34,51,52,53]. The flaws of conventional testing procedures can also be eliminated using these models [54,55]. Realistic predictions for concrete properties can be made using advanced machine learning techniques and regression methods. The current study was conducted to extract the most effective computational technique/model to accurately forecast the compressive strength of concrete.

## 2. Research Significance

Incorporating recycled aggregates (RAs) in conventional and self-consolidating concretes for various civil engineering construction and research projects/studies has been gaining attention globally. A wide range of options is available in reusing materials for building construction due to the utilization of RA as a concrete ingredient. Incorporating RA in concrete is an effective solution to control the waste problem. None of the research from almost the last 50 years has shown the unsuitability of RAs for structural members. Accordingly, the current study is focused on exploring the compressive behaviour of RCAC. A precise forecasting model can be beneficial for researchers and engineers to assess the RAC mechanical characteristics and can conserve cost and time with laboratory experimentations. The mix design for RAC, which includes satisfactory strength parameters, reduced cost, and CO_2_ emissions, needs to be optimized for execution in construction projects. The optimization of an RAC mix can be attained with the help of developed AI models. The main aim of this research was to develop models that provide realistic predictions for concrete properties using advanced machine learning techniques. The current study extracted the most effective computational technique/model to forecast the compressive strength of RCAC accurately. Furthermore, an extensive literature review was conducted to extract a comprehensive and reliable experimental database with a wide variety of mix proportions for RCAC compressive properties. The economical and efficient design for durable structures can be achieved by the accurate prediction of concrete properties, which will ultimately reduce the time required for selecting appropriate materials and repairs and the consumption of resources. The proposed prediction methods can also enable researchers in the civil engineering field to design new materials smartly. An enhanced explanation of ML models, with thorough insight into the importance of all the corresponding features with the help of SHAP analysis, is also provided. The performance of SHAP analysis on existing artificial intelligence models is the current research’s innovation. This could improve civil engineers’ ability to use artificial intelligence approaches in the construction industry. The step-by-step laboratory method, which includes casting specimens, curing for a certain period, and testing, remains a source of concern in terms of cost and time.

## 3. Brief Methodology for Prediction

### 3.1. SVM Bagging and AdaBoost Model

SVM is a supervised learning technique with associated learning algorithms to analyse regression analysis and classification data. The support vector machine model technique classifies similar data points together and maps them onto a clear vector (line or plane), with possible wide gaps. The same space is used for mapping new examples as per the prediction of belonging to a category defined by the side of the vector. In the current study, the SVM model was majorly utilised to explore the reduction in concrete strength. The ML model was constructed by applying a new optimisation algorithm.

The determination of reduced concrete strength was conducted using the SVM model, keeping in mind the compound effect of various factors. The selection of factors for the SVM model was carried out using an optimisation algorithm. The particular algorithm is as follows:

#### 3.1.1. Determination of the Training Dataset

In Equation (1), ${\left\{X_{i},{\;Y}_{i}\right\}}_{i=1},{\;X}_{i}\in R^{n}$ is a database with group i concrete influential strength factors; $Y_{i}\in R^{n}$ is the predicted output (i.e., group i post-degradation concrete strength). All of this data are from the training database.

(1) $$T\;=\left\{\left(x_{1},y_{1}\right),\;\ldots \;,\;\left(x_{1},y_{1}\right)\right\}\in {\left(X\times Y\right)}^{1}$$

#### 3.1.2. Choosing Kernel Function

In the current study, the radial basis function (RBF) was used, as given in Equation (2):

(2) $$K\left(X,X_{i}\right)=\exp\left(-\mathrm{gamma}∥X-X_{i}∥{}^{2}\right)$$

As per the principle of structure optimisation, the following expression can be used for the regression optimisation goal:

$$\max^{\alpha \alpha }\overset{I}{\underset{i=1}{\sum }}y_{i}\left(\alpha_{i}^{*}-\alpha_{i}\right)-\epsilon \left(\alpha_{i}^{*}+\alpha_{i}\right)-\frac{1}{2}\overset{I}{\underset{i=1}{\sum }}\times \overset{I}{\underset{j=1}{\sum }}\left(\alpha_{i}^{*}-\alpha_{i}\right)\left(\alpha_{j}^{*}-\alpha_{j}\right)K\left(X_{i},X_{j}\right)$$

$$\mathrm{subject}\;\mathrm{to}\;\sum_{i=1}^{I}\left(\alpha_{i}^{*}-\alpha_{i}\right)=0,0\leq \alpha_{i},\alpha_{i}^{*}\leq c,\;i=1,\;2,\ldots \;,I$$

where ∈ is the deviation, g (gamma) is a kernel function, and c is the penalty parameter.

**Figure 2:**
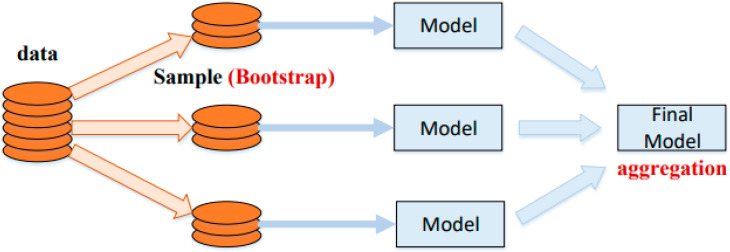
Bagging algorithm process [57].

**Figure 3:**
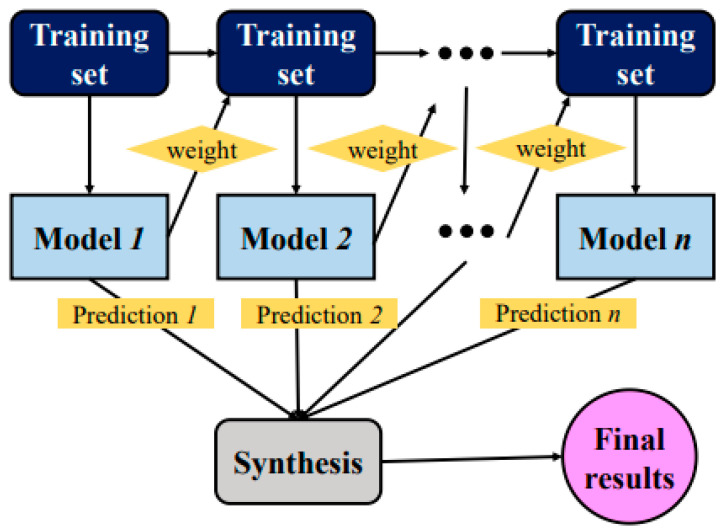
AdaBoost algorithm process [58].

The application of ensemble techniques optimises machine learning and data mining operations. In addition, the said methods are helpful for mitigating unnecessary training data concerns by pooling and merging various sub-models of components. A large amount of classifier components/sub-models/(1, 2, …, M) can be generated by carefully altering the training data, ultimately leading to a productive learner. On the other hand, the average combined methods on verified sub-models can be used to build an optimised prediction model. In parallel with the benefit collation and bootstrap resampling technique, Bagging is one of the most widely used ensemble modelling techniques. The first training dataset (bootstrap testing w.r.t size of the training set) replaces the component models. Most of the dataset points can frequently appear in product models, whereas others may not appear. Consequently, the average of all the outputs of the component model is taken as its final output [56]. The methodical flow chart for Bagging is shown in Figure 2. Figure 3 depicts the same for the AdaBoost algorithm. A cumulative model was generated using the boosting method, which produced component development of high precision w.r.t individual model. In the meantime, the weighted averages of the respective sub-models were used in the final model from the boosting positions of dependent sub-models. In the current study, SVM boosting and Bagging were employed to predict concrete mechanical properties.

### 3.2. Gradient Boosting and XG Boost Models

A substitute for regression methods can be achieved by a statistical technique named regression tree [59]. In the regression tree technique, the entire dataset is split into two or more uniform sets to build a model. Upon the termination of the splitting process, a node is named a terminal node. A single value is termed a decision node upon which each node is split into sub-nodes. The recurring binary splitting is used to build a regression tree model with input considerations and a response parameter (Equation (3)).

(3) $$R_{1}\left(j,\;s\right)=\left\{{X|X}_{j}\leq s\right\}{\;\mathrm{and}\;R}_{2}\left(j,s\right)=\left\{{X|X}_{j}>s\right\}$$

where s and j are the splitting point and variables, respectively. Further, s and j are used for achieving the most uniform splitting group.

**Figure 4:**
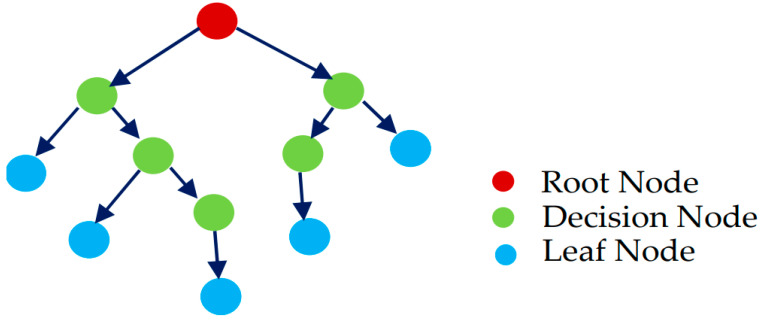
Structure of a regression tree [60].

The splitting point and variable that were selected are as follows:

$$\sum_{X_{i}\in R_{1}\left(j,s\right)}{\left(y_{i}-{ŷ}_{R_{1}}\right)}^{2}-\sum_{X_{i}\in R_{2}\left(j,s\right)}{\left(y_{i}-{ŷ}_{R_{2}}\right)}^{2}$$

where ${ŷ}_{R_{1}}\mathrm{and}\;{ŷ}_{R_{2}}$ are the mean values of the corresponding group responses. Upon building a regression tree, the new observation prediction relies on splitting the observed value from the root to the terminal node, as depicted in Figure 4.

**Figure 5:**
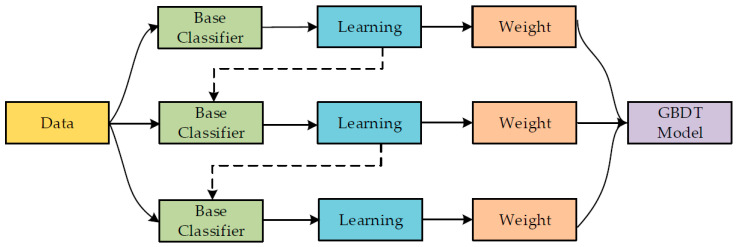
Gradient boosting training process [62].

This ensemble method for classification and regression was proposed by Friedman [61]. The gradient boosting method is similar to other boosting techniques but limited to regression only. In this technique, each training set iteration is selected randomly and validated by the base model, as represented in Figure 5. The execution accuracy and speed of gradient boosting can be enhanced by training data sub-sampling, which aids in avoiding the issue of overfitting. The smaller fraction of training data is obtained with a higher speed of regression for fitting lower model data at every iteration. The shrinkage rate and n-trees tuning factors are needed in gradient boosting regression; however, the n-trees denote the number of grown trees. Here, an overly small n-tree value was avoided, and the shrinkage factor, called the learning rate, was applicable to each expansion tree.

Chen and Guestrin [63] proposed the extreme gradient boosting (XG Boost) algorithm, which is considered an authentic tool for researchers in the data science field due to the effective tree-based ensemble learning algorithm. Gradient boosting architecture, i.e., the application of different functions for the estimation of results using Equation (4), is the basis of XG Boost [61].

(4) $${\bar{y}}_{i}=y_{i}^{0}+ɳ\sum_{K=1}^{n}f_{k}\left(U_{i}\right)$$

where predicted output is shown by ${\bar{y}}_{i},$ with ith data and $U_{i}$ as a parameter vector; n shows the number of estimators in correspondence with the independent tree structures against every $f_{k}$, where the range of k is from 1 to n; and $y_{i}^{0}$ is the main hypothesis (mean of the original factors in the training dataset). η depicts the learning rate to enhance the model performance, along with the connection of additional trees to avoid overfitting. One major conflict in ML is developing a model with the least overfitting. The training phase is complementarily evaluated in the XG Boost model.

**Figure 6:**
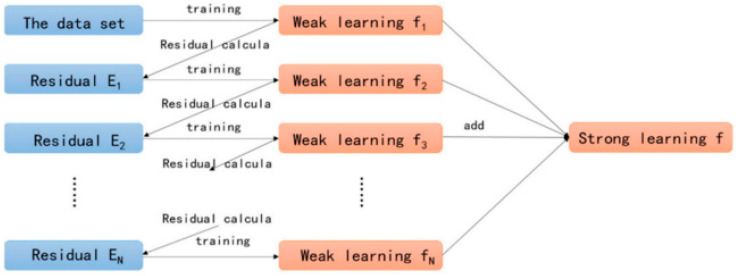
Extreme gradient boosting (XG Boost) algorithm structure [64].

As per Equation (4), at the kth level, the kth estimator is connected to the model, and the forecasting of kth $y_{i}^{-k}$ is determined through the predicted output $y_{i}^{-\left(k-1\right)}$ in a further step. The respectively developed f_k_ against the kth complementary estimator is provided in Equation (5).

(5) $$y_{i}^{-k}=y_{i}^{-\left(k-1\right)}+ɳf_{k}$$

where f_k_ depicts the weight of leaves, developed by minimising the kth tree objective function (Equation (6)).

(6) $$f_{\mathrm{obj}}=\gamma Z+\sum_{a=1}^{Z}\left[g_{a}\omega_{a}+\frac{1}{2}\left(h_{a}+\lambda \right)\omega_{a}^{2}\right]$$

where the number of leaf nodes is denoted by Z, complexity factor by c, constant coefficient by λ, and the weight (i.e., 1–Z) of a leaf by $\omega_{a}^{2}$. λ and c are controlling factors applied to improve the model in terms of avoiding overfitting. $h_{a}$ and $g_{a}$ are the sum factors for the whole dataset linked to a previous and an initial loss function gradient leaf, respectively. For building the kth tree, a leaf is further bifurcated into multiple leaves. Gain parameters are used to apply such a system, as given in Equation (7).

(7) $$G=\frac{1}{2}\left[\frac{O_{L}^{2}}{P_{L}+\lambda }+\frac{O_{R}^{2}}{P_{R}+\lambda }+\frac{{\left(O_{L}+O_{R}\right)}^{2}}{P_{L}+P_{R}+\lambda }\right]$$

where the gain parameters are denoted by G, right and left leaf, PR and OR, and P_L_ and O_L_, respectively. The division criteria are generally assumed when approximating the gain parameter to zero. λ and c are controlling factors that are indirectly dependent on the gain parameters. For instance, the gain parameter can be considerably decreased by a more significant regularisation parameter, ultimately preventing the leaf convolution process. However, the model performance for adopting training data would also be reduced by this. The basic level-wise structure of the XG Boost tree model is shown in Figure 6.

### 3.3. Model Interpretability with SHAP

The learning ability of ML models from known data to predict responses in unexplored regions has captured all efforts for the development of robust estimation tools. However, in most ML modelling techniques, their suffering from high complexity and lesser interpretability is common. Game theory with Shapley values enhance explanations for models. This is called SHapley Additive exPlanations (SHAP) [65], and they are applied to evaluate the importance of different features in a respective model [45,66]. For the feature importance if “j” for “f” as a model’s output in SHAP, $\phi^{j}\left(f\right)$ is assigned a weight against the summation of the contribution of the feature for the model’s outcome $f\left(x_{i}\right)\;$across all potential feature combinations [67]. Equation (8) shows the expression for $\phi^{j}\left(f\right)\;$as follows:

(8) $$\phi^{j}\left(f\right)=\sum_{S\subseteq \left\{x^{1},\ldots \ldots ,x^{p}\right\}/\left\{x^{j}\right\}}\frac{\left|S\right|!\left(p-\left|S\right|-1\right)!}{p!}\left(f\left(S⊔\left\{x^{j}\right\}\right)-f\left(S\right)\right)$$

where

$x_{j}$ = feature *j*,

*S* = feature subset, and

*p* = feature number in the model.

In this method, feature contribution is explored by computing the estimation errors during a specified feature value disturbance. The sensitivity of the estimation error is used to assign a weight to the importance of the feature during the disturbance of the feature value. The performance of the trained ML model is also described with the help of SHAP. It utilises a further feature attribution technique, such as the addition of input parameters for describing a model to be considered with the output model. For example, a model’s input parameters are $x_{i},$ where i is between 1 and k, where k depicts the number of input parameters and h ($x_{s}$) depicts the explanation model with $x_{s}$ as the simplified input. However, Equation (9) is applied to show a genuine model f(x):

(9) $$f\left(x\right)=h\left(x_{s}\right)=\emptyset_{0}+\sum_{i=1}^{p}\emptyset_{i}x_{s}^{i}$$

where

p = input feature number and

$\emptyset_{0}$ = no information constant.

**Figure 7:**
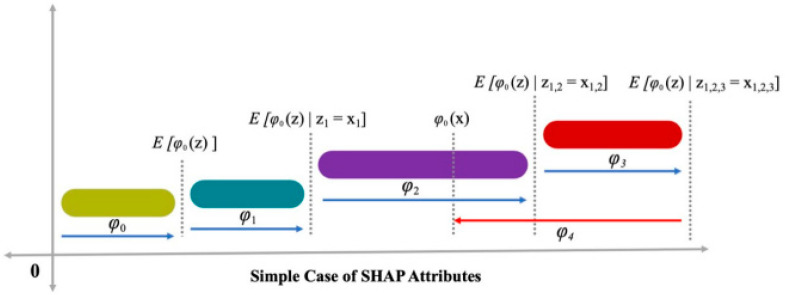
Attributes of SHAP [68].

There is a correlation between the mapping function, i.e., $x=m_{x}\left(x_{s}\right),$ and both the x and $x_{s}$ inputs. Lundberg and Lee [45] presented a figure (i.e., Figure 7) to demonstrate Equation (9), which enhances the estimation value, i.e., (h ()), by $\emptyset_{0},\;\emptyset_{1},\;and\;\emptyset_{3};$ a decrement of $\emptyset_{4}$ is also observed. Equation (9) has a single solution comprising three desired characteristics: local accuracy, consistency, and missingness. Local accuracy ensures the summation of attribution features as an outcome function that contains a model requirement for matching the “f” outcome for $x_{s}$ as simplified input, where $x=m_{x}x_{s}$ depicts the attainment of local accuracy. Missingness ensures no importance, i.e., $\emptyset_{i}=0,$ is implied when $x_{s}^{i}=0$. There is no reduction in attribution as ensured by consistency, which is assigned to the concerned feature because of changes in features of higher impact.

## 4. Data Collection

### Dataset Description

**Figure 8:**
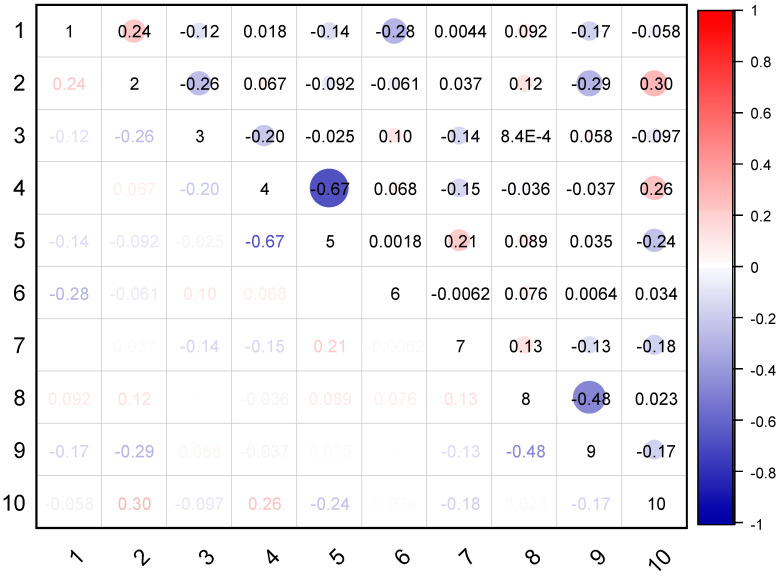
Kendall’s correlation.

**Figure 9:**
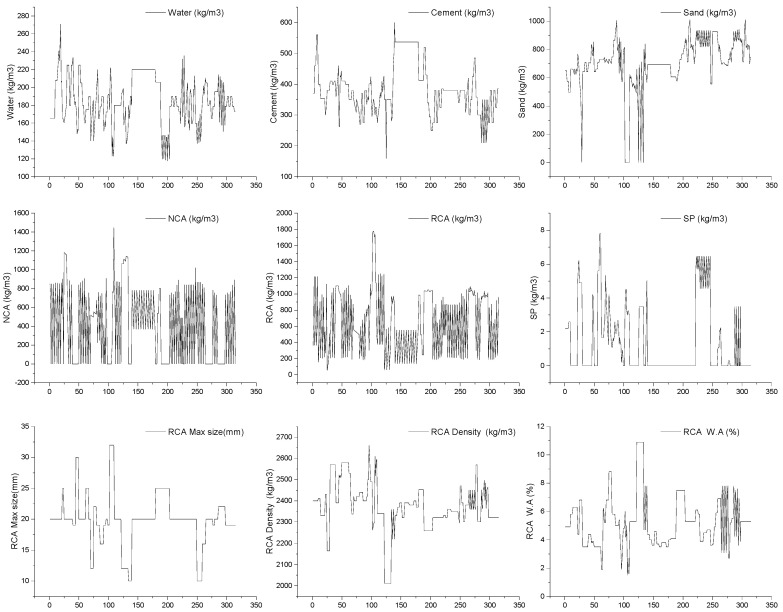
Input parameter distribution.

RCAC has different mechanical properties than conventional natural aggregate concrete (CNAC). Recycled aggregate (RA) has lesser density and is more capable of WA when compared to those of natural aggregates [69], depicting the worse mechanical properties and workability of RCAC with respect to CNAC [70]. As previously discussed in the literature [71], the deterioration of RCAC mechanical properties increases with increasing RA content. Accordingly, in addition to the basic ingredients of conventional concrete (i.e., water, sand, natural aggregate, cement, and super-plasticizer), four RA properties (i.e., RA content, density, WA, and maximum size) are also being taken as input parameters. The prediction of compressive strength (σ) as an output was made. From the previous literature, 317 RCAC mixtures were collected to be considered as input and output parameters for developing the dataset, as shown in Table S1. Table 1 shows the statistics of the input and output parameters (only CS). Figure 8 depicts Kendall’s correlation matrix of the input and output parameters (only CS). The weak correlations (i.e., less than 0.5) show that multicollinearity issues are not caused by these variables [72]. The database input variable curve is shown in Figure 9.

## 5. Results and Discussion

### 5.1. SVM-AdaBoost Models

**Figure 10:**
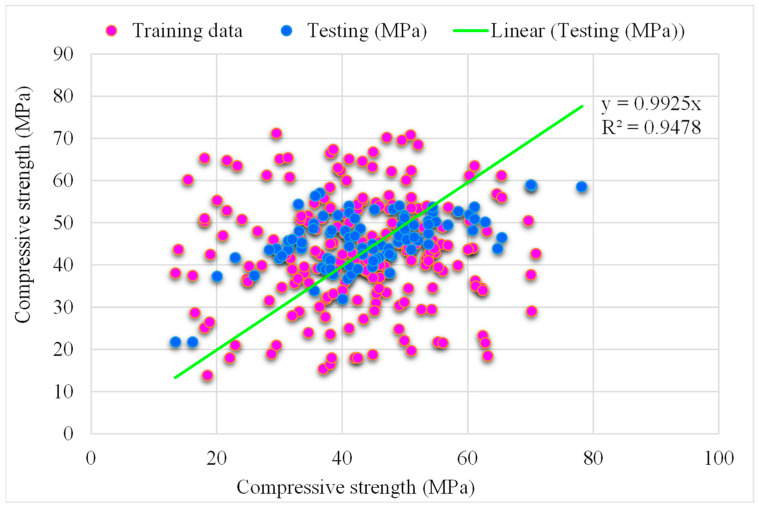
Experimental and SVM-AdaBoost predicted results.

**Figure 11:**
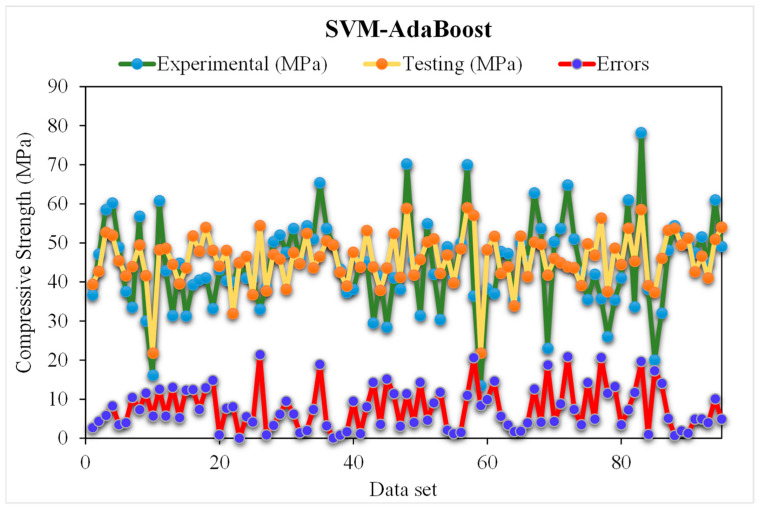
Distribution of experimental and SVM-AdaBoost predicted values with errors.

Figure 10 depicts the predicted and experimental SVM-AdaBoost outcomes for RCAC compressive strength. The SVM-AdaBoost correlation coefficient (R^2^) value is 0.94 and shows more accurate and precise results. The higher accuracy is because of the ensemble machine learning models that used the results of optimised models for predicting the compressive strength of RCAC based on 20 sub-models. The distribution of the SVM-AdaBoost predicted and experimental values, along with the error values for RCAC, are demonstrated in Figure 11. Please note that only the testing data are provided in Figure 11. It may be observed that in the error data, 42% of the values are less than 5 MPa, 26% range from 5 to 10 MPa, and 31% of the values are above 10 MPa. However, only a few error values are greater than 20 MPa, and most of them are less than 10 MPa (69%). The lower error and higher R^2^ values exhibit the higher precision of the SVM-AdaBoost algorithm.

### 5.2. SVM-Bagging Models

**Figure 12:**
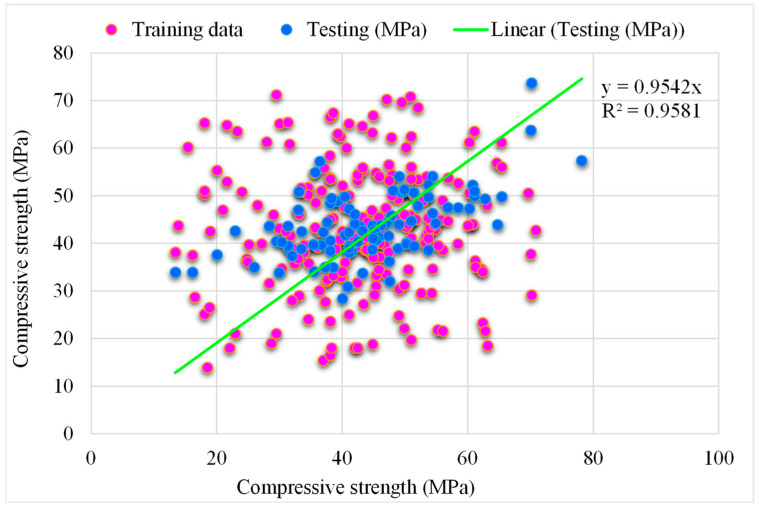
Experimental and SVM-Bagging predicted results.

**Figure 13:**
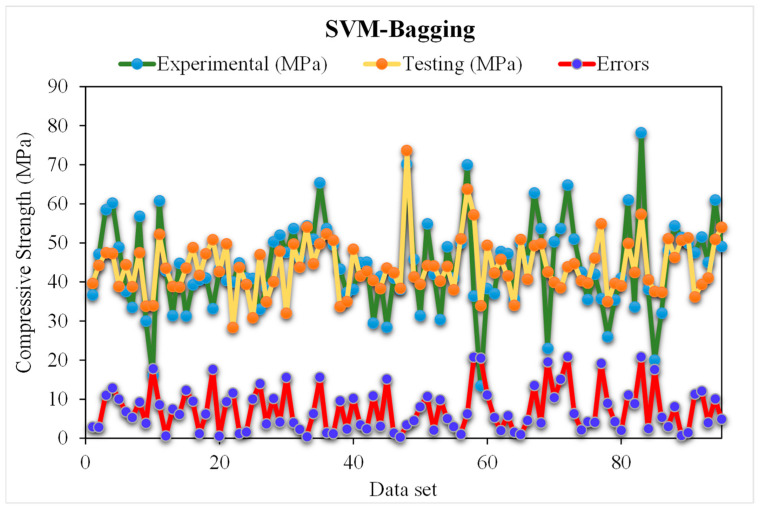
Distribution of experimental and SVM-Bagging predicted values with errors.

Figure 12 depicts the SVM-Bagging predicted and experimental outcomes for RCAC. An R^2^ of 0.95 was obtained in the case of this model, which represents more satisfactory outcomes than those of SVM-AdaBoost. Figure 13 illustrates the distribution of the SVM-Bagging estimated and experimental values of RCAC strength, with errors. Out of the error values, 44% are less than 5 MPa, 24% are from 5 to 10 MPa, and the remaining 31% are more than 10 MPa. The R^2^ and error values for RCAC strength are precise as compared to those of SVM-AdaBoost. However, only a few error values are greater than 10 MPa and most of them are less than 10 MPa (69%). On the other hand, a low error rate for the compressive strength of RCAC was observed in the SVM-Bagging models compared to that of AdaBoost.

### 5.3. DT-Gradient Boosting Models

**Figure 14:**
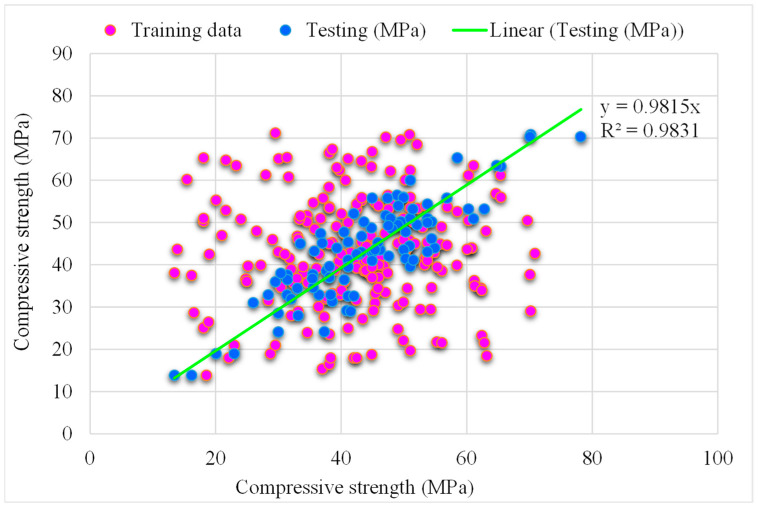
Experimental and gradient boosting predicted results.

**Figure 15:**
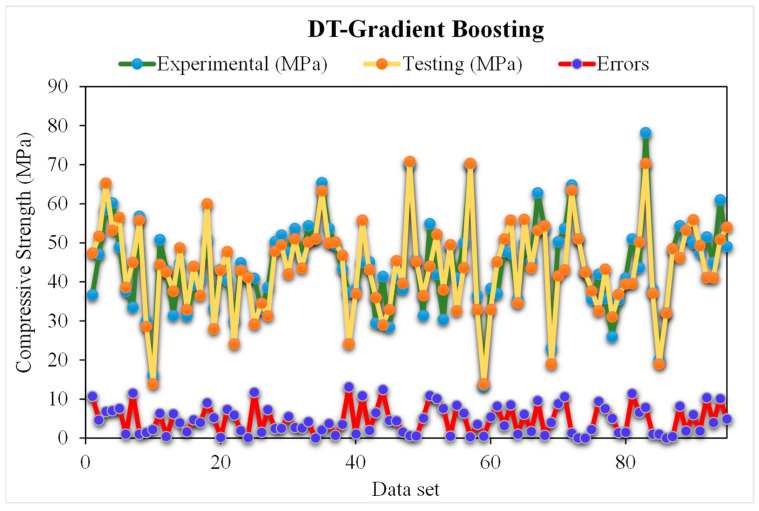
Distribution of experimental and gradient boosting predicted values with errors.

Figure 14 presents the estimated gradient boosting and experimental outcomes for RCAC strength. The R^2^ for AdBSVM was determined to be 0.98 and shows more accurate outcomes compared to the ensemble SVM model. Figure 15 shows the gradient boosting value estimated, along with the errors and experimental result distribution for RCAC strength. It may be observed that among the error values, 57% are less than 5 MPa, only 16% are more than 10 MPa, and the remaining 25% are between 5 and 10 MPa. However, only a few error values are greater than 20 MPa, and most of them are less than 10 MPa (83%). The higher R^2^ values and lower error values exhibit this model’s better accuracy compared to that of other ensemble SVM models.

### 5.4. DT-Extreme Gradient Boosting (XG-Boost) Models

**Figure 16:**
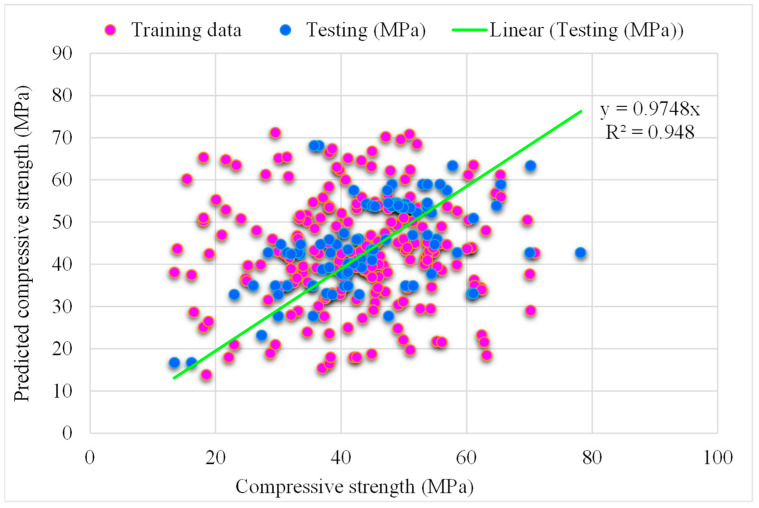
Experimental and extreme gradient boosting predicted results.

**Figure 17:**
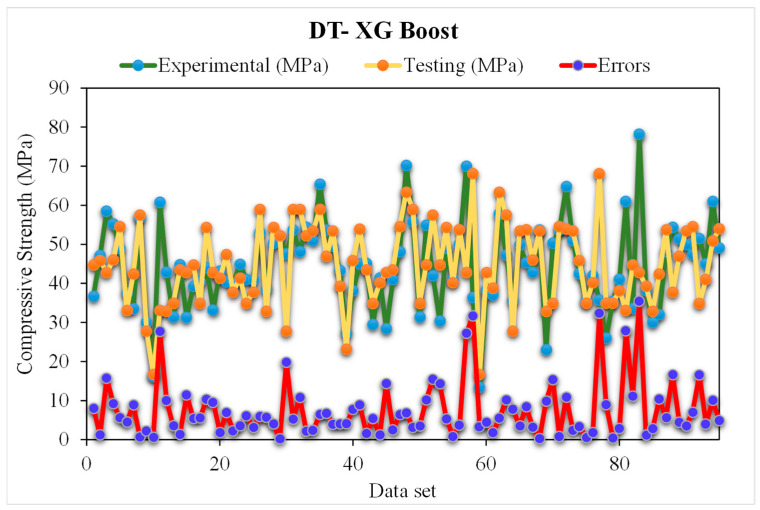
Distribution of experimental and extreme gradient boosting predicted values with errors.

Figure 16 shows the XG-Boost estimated and experimental outcomes of RCAC strength. In this model, the R^2^ was 0.94, which depicts more satisfactory results. However, the anticipated compressive strength results of RCAC with XG-Boost are not as good compared to other models. The distribution of the XG-Boost estimated and experimental values for RCAC strength, along with errors, is shown in Figure 17. Out of all the error values, 46% are under 5 MPa, 26% are above 10 MPa, and 27% are between 5 and 10 MPa. The R^2^ and error values for RCAC strength have lower precision compared to the other ensemble models. On the other hand, a lower error rate for the compressive strength of RCAC was observed in DT-Gradient Boosting models compared to that of all other models. However, the R^2^ and error values obtained from all of the considered ensemble machine learning models are within an acceptable range, indicating better prediction results. Thus, this finding implies that ensemble machine learning models (DT-Gradient Boosting) may estimate results with more precision compared to other ensemble models.

## 6. Model Comparison and Performance Evaluation

Model performance is conventionally predicted using a wide variety of performance criteria for choosing a model with higher accuracy, leading towards prediction framework steadiness. Accordingly, a different performance criterion was proposed in the current study, as follows:

The regression survey is mainly dependent on a correlation coefficient (R^2^). The calculation for R^2^ is provided in Equation (10).

(10) $$R^{2}=\frac{\sum_{i=1}^{n}\left(X_{i}-\bar{X}\right)\left(Y_{i}-\bar{Y}\right)}{\sqrt{\sum_{i=1}^{n}{\left(X_{i}-\bar{X}\right)}^{2}{\left(Y_{i}-\bar{Y}\right)}^{2}}}$$

The mean absolute error (MAE) is an important metric to determine a model’s performance. The calculation of MAE is shown in Equation (11).

(11) $$\mathrm{MAE}=\frac{1}{n}\sum_{i=1}^{n}\left|x_{i}-x\right|$$

The square root of the MSE is taken as the root mean square error (RMSE) and is also considered a considerable metric for mathematical modelling. The calculations for the RMSE are given in Equation (12).

(12) $$\mathrm{RMSE}=\sqrt{\frac{\sum_{i=1}^{n}{\left(X_{i}-Y_{i}\right)}^{2}}{n}}$$

**Figure 18:**
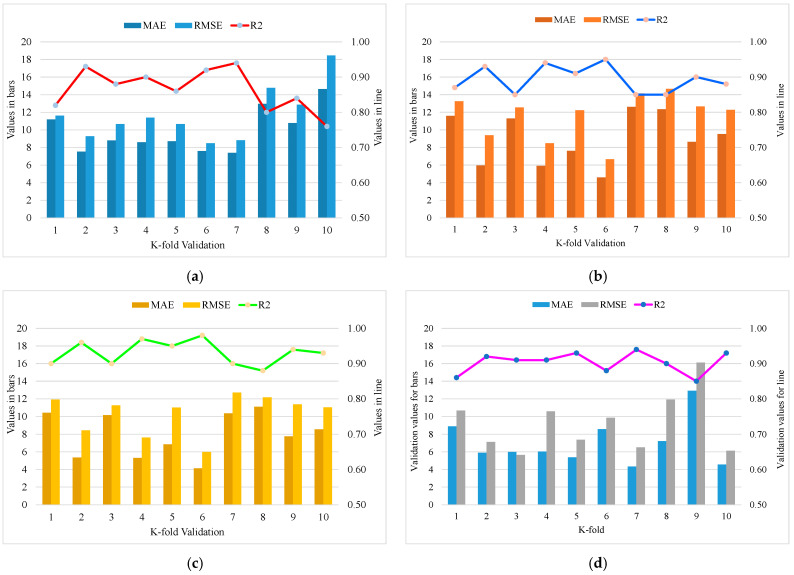
K-fold cross-validation. (**a**) SVM-AdaBoost; (**b**) SVM-Bagging; (**c**) DT-Gradient Boosting; (**d**) DT- XG-Boost.

Statistical methods are usually employed for assessing the model’s performance [28,73,74,75], as shown in Equations (10)–(12). For execution, the assessment of a model’s validity is done with the help of the k-fold cross-validation approach. Greater R^2^ values with lower error (MAE and RMSE) values represent higher accuracy of the models. The data are usually split into ten sub-groups for random dispersion in k-fold cross-validation, and the method is repeated 10 times to attain satisfactory outcomes. The statistical analysis results of all models are provided in Table 2. The R^2^ values for the optimal SVM-AdaBoost, SVM-Bagging, DT-Gradient Boosting, and DT- XG Boost models are 0.94, 0.95, 0.98, and 0.94, respectively (Figure 18). It was found that the R^2^ of DT-Gradient Boosting was higher compared to the other considered models with lower error values in the case of RCAC compressive strength.

**Figure 19:**
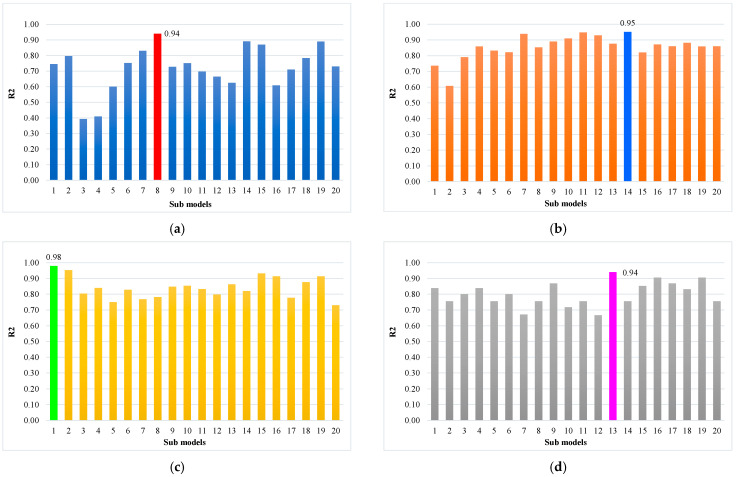
Sub-models. (**a**) SVM-AdaBoost; (**b**) SVM-Bagging; (**c**) DT-Gradient Boosting; (**d**) DT-XG-Boost.

Ensemble machine learning techniques were evaluated in this study to predict the accurate and precise results for the compressive strength of RCAC with mixed ingredients. The output of DT-Gradient Boosting presents more precise predicted results for RCAC compressive strength, with an R^2^ value of 0.98, as shown in Figure 19. The ensemble machine learning models exhibited better results because 20 sub-models were generated for predicting the value of compressive strength, and an optimised model was used. Therefore, the ensemble DT-Gradient Boosting models showed high accuracy with low error compared to all other models.

## 7. Enhanced ML Models Explain Ability

**Figure 20:**
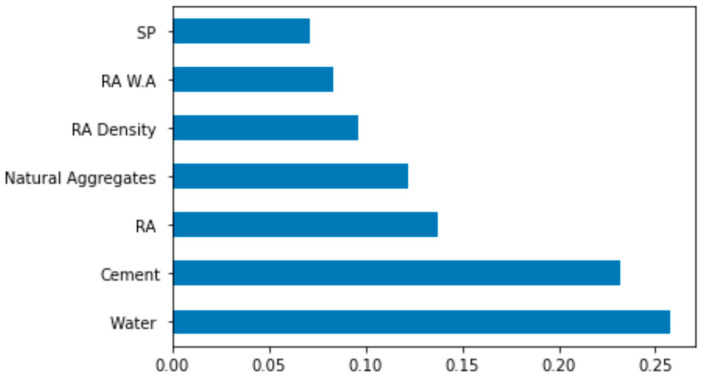
Importance of features.

An in-depth explanation is provided in the current paper regarding the use of the ML model and the dependencies/interactions of all taken features. The SHAP tree explainer is employed initially across a whole database to demonstrate an enhanced global depiction of the feature influences by combining local SHAP explanations. A SHAP method called “TreeExplainer” is applied [76]. The inner composition of a model, which is tree-based, is explored using this technique, obtaining the sum of a calculation set linked to the leaf node of a tree model that leads to low-order complexity [76]. This section analyses the model for RCAC compressive strength by employing SHAP. The SHAP values and feature correlation for RCAC compressive strength are presented in Figure 20.

It was observed that the highest SHAP value to estimate RCAC compressive strength was for water content. The cement content feature had the second highest SHAP value. So, it can be summarised that water content influences the output, i.e., the compressive strength, followed by the cement content and recycled and natural aggregates. The higher the influence of the water content might be, the higher the WA property of the RA.

**Figure 21:**
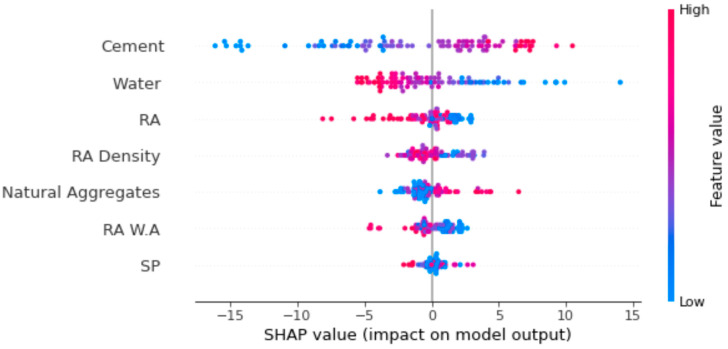
SHAP plot.

A violin plot of the SHAP values for all the considered features is shown in Figure 21 for the prediction of RCAC compressive strength. In this plot, each feature value is represented by a unique colour, and the respective *x*-axis value (i.e., SHAP value) depicts the contribution of the outcome. For example, for water content as an input feature, on the right side of the axis, lower values are represented by blue-coloured points, and on the left side of the axis, high values for water content are represented by red-coloured values. The 12 SHAP values, as red points, indicate that higher cement content enhances the compressive strength of RCAC, depicting its positive influence. However, the water content shows a negative influence, representing its inverse relationship with the compressive strength of RCAC. Similarly, recycled aggregate negatively influences the compressive strength of RCAC, as its density, although slight, is in inverse correlation with the compressive strength of RCAC. However, natural aggregates positively influence the compressive behaviour of RCAC due to its low WA capacity. It may be noted here that the WA capacity of aggregates also has an inverse relationship with RCAC compressive strength. Similarly, in the case of super plasticisers, the trend shows that with increasing content, the compressive strength decreases, as depicted in Figure 21. This prediction is subjected to the database used in the current research, and outcomes with higher precision could be attained with more data points.

**Figure 22:**
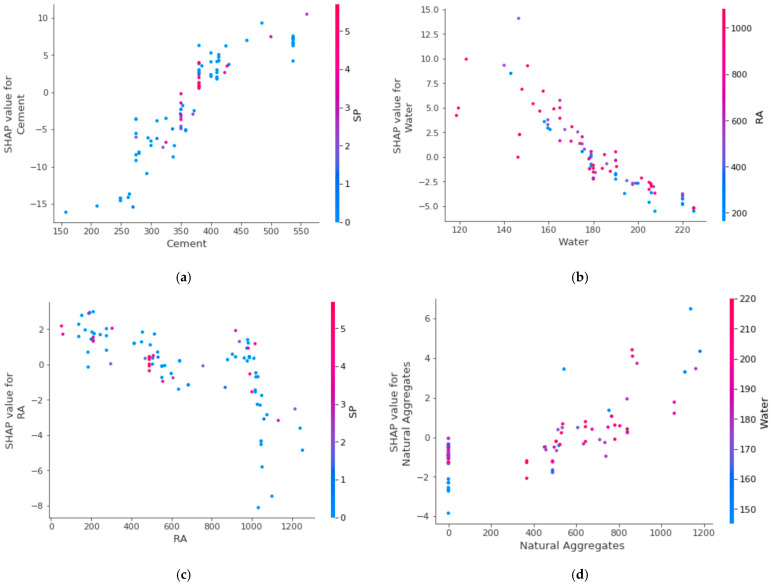
Relationship of various parameters: (**a**) cement; (**b**) water; (**c**) RA; and (**d**) natural aggregate.

Figure 22a shows the cement feature interaction. It can be noted from the plot that cement directly influences RCAC compressive strength. Further, a considerable cement interaction with SP was observed, which results in a positive influence of cement on compressive strength. The inverse influence of water on RCAC compressive behaviour is noted in Figure 22b. The water feature shows higher interaction with RA. This indicates a decrease in the compressive strength with an increase in the content of water. Therefore, water appears to have a negative impact on the compressive strength of RCAC, illustrating an inverse relationship. The recycled coarse aggregate feature interaction is plotted in Figure 22c, which is an inverse correlation. A higher WA capacity tends toward a negative influence of recycled coarse aggregates on RCAC compressive strength. However, in contrast, the positive influence of natural aggregates on the compressive behaviour of RCAC can be seen in the plot of the natural coarse aggregates feature in Figure 22d.

## 8. Conclusions

The levels of accuracy of the existing models in the literature are still limited to forecasting the RCAC properties. Accordingly, this research gap was addressed by developing dependable analytical tools for predicting and interacting with various parameters in the current study. In this study, the compressive property of RCAC was evaluated using four advanced ensemble machine learning techniques, i.e., SVM-AdaBoost, SVM-Bagging, DT-Gradient Boosting, and DT- XG Boost. Furthermore, all the models were validated through statistical checks. The conclusions of the conducted analytical study are as follows:

1. An optimised model was developed to forecast the RCAC compressive property with considerably enhanced accuracy among all ensemble machine learning models.
2. The suitability of the developed models to be used in the pre-design of RCAC is highly favourable due to the higher level of model accuracy to forecast the compressive strength of RCAC.
3. The DT-Gradient Boosting model outperformed all other models in terms of prediction with greater R^2^ and lower error levels. SVM-AdaBoost, SVM-Bagging, DT-Gradient Boosting, and DT-XG Boost models had R^2^ values of 0.94, 0.95, 0.98, and 0.94, respectively. However, the ensemble model results of the DT-Gradient Boosting model, followed by SVM-Bagging, are also acceptable.
4. The mean square error values of the SVM-AdaBoost, SVM-Bagging, DT-Gradient Boosting, and DT-XG Boost models were 7.7, 7.4, 4.7, and 7.7 MPa, respectively. DT-Gradient Boosting obtained predictions with higher precision and a low error rate for the compressive strength of RCAC.
5. The k-fold cross-validation technique and statistical analysis revealed satisfactory DT-Gradient Boosting and SVM-Bagging outcomes. These tests also showed that the DT-Gradient Boosting model outperformed the SVM-AdaBoost, SVM-Bagging, and DT- XG Boost models.
6. Upon comparison, it is concluded that the DT-Gradient Boosting model with a regression coefficient (R^2^) closer to 1 is preferable to all other models.
7. The water content has the highest impact on compressive strength prediction, followed by cement and recycled coarse aggregates, as shown by the importance of features. After these features, natural aggregates and the RA density have comparable influence.
8. The feature interaction plot using SHAP analysis shows that with increasing water and recycled coarse aggregates content in RCAC, the compressive strength decreases.
9. The consumption of natural resources and energy to process these materials for the manufacturing of concrete leads to environmental degradation with CO_2_ emissions. Further, landfill contamination due to construction and demolition waste also impacts the environment negatively. Hence, incorporating RA in concrete can significantly play a role in its environment-friendly development by reducing landfill pollution.

There are some new prediction approaches [77,78,79,80,81,82,83,84,85], such as adaptive neuro-fuzzy inference system (ANFIS), deep learning (DL), marine predators algorithm (MPA), pure random orthogonal search (PROS), artificial neural networks (ANNs), genetic algorithm (GA), and particle swarm optimization (PSO) method, etc. These approaches can better reveal the strength of structures in a more unbiased way, which should be studied in the future. In addition, this research was limited to compressive strength prediction with nine input parameters and did not consider other factors. Indeed, proper database and testing must be applied, as they are vital elements for engineering applications. This study was based on a wide range of data sets with nine input variables; however, the database and more input parameters, such as specimen size, curing age, etc., need to be generated in the future to obtain better results for the employed models.

## Figures and Tables

**Table 1:** Basic input and output parameters (CS only).

SR #	Parameters	Mean	Standard Error	Median	Mode	Range	Minimum	Maximum
	Input							
1	Water (kg/m^3^)	185	1.50	180	220	153	118	271
2	Cement (kg/m^3^)	385	4.59	380	380	442	158	600
3	Sand (kg/m^3^)	703	10.37	706	693	1010	0	1010
4	Natural Aggregates (kg/m^3^)	412	20.96	489	0	1448	0	1448
5	RA (kg/m^3^)	629	20.26	543	138	1726	52	1778
6	SP (kg/m^3^)	1.35	0.12	0	0	8	0	8
7	RA Max. Size (mm)	19.90	0.23	20	20	22	10	32
8	RA Density (kg/m^3^)	2373	6.50	2370	2320	651	2010	2661
9	RA WA (%)	5.25	0.10	5	5	9	2	11
	Output							
10	CS (MPa)	43.56	0.71	43.3	41	65	13	78

**Table 2:** Statistical check of SVM, AdBSVM, and BSA models.

Techniques	MAE (MPa)	RMSE (MPa)	R^2^
SVM-AdaBoost	7.7	9.5	0.94
SVM-Bagging	7.4	9.3	0.95
DT-Gradient Boosting	4.7	5.9	0.98
DT-XG Boost	7.7	10.5	0.94

## Data Availability

The data used in this research are properly cited and reported in the main text.

## Supplementary Materials

The following supporting information can be downloaded at: https://www.mdpi.com/article/10.3390/ma15155207/s1, Table S1: Database for recycled aggregate concrete used in modelling. References [86,87,88,89,90,91,92,93,94,95,96,97,98,99,100,101,102,103,104,105,106,107,108,109,110,111,112,113,114,115,116,117] are cited in the Supplementary Materials.
