# Supplementary material for: Use of Artificial Intelligence for Predicting Parameters of Sustainable Concrete and Raw Ingredient Effects and Interactions
Source: Materials (Basel). 2022 Jul 27;15(15):5207. doi: 10.3390/ma15155207 (PMC9369900; doi:10.3390/ma15155207)
Supplement: Supplementary file 1 [file materials-15-05207-s001.zip › materials-1771857-supplementary.pdf]

Table S1. Database for recycled aggregate concrete used in modeling.

Ref.	Water (kg/m³)	Cement (kg/m³)	Sand (kg/m³)	NCA (kg/m³)	RCA (kg/m³)	SP (kg/m³)	D_{max_RCA} (mm)	ρ_{RCA} (kg/m³)	W_{RCA} (%)	UCS (MPa)
[1]	165	370	650	850.5	364.5	2.22	20	2400	4.9	50.6
	165	370	650	607.5	607.5	2.22	20	2400	4.9	50.8
	165	370	650	0	1215	2.22	20	2400	4.9	50.2
	165	460	575	850.5	364.5	2.22	20	2400	4.9	60.8
	165	460	575	607.5	607.5	2.22	20	2400	4.9	61.2
	165	460	575	0	1215	2.22	20	2400	4.9	60.2
	165	560	495	850.5	364.5	2.59	20	2400	4.9	70.2
	165	560	495	607.5	607.5	2.59	20	2400	4.9	70.8
	165	560	495	0	1215	2.59	20	2400	4.9	70
[2]	207.6	400	662	863	153	0	20	2410	5.8	38.1
	207.6	400	662	697	298	0	20	2410	5.8	37
	207.6	400	662	383	573	0	20	2410	5.8	35.8
	207.6	400	662	0	903	0	20	2410	5.8	34.5
[3]	217	353	660	861	209	0	20	2330	6.3	44.9
	229	353	647	527	513	0	20	2330	6.3	44.7
	241	353	625	0	993	0	20	2330	6.3	46.8
	230	353	661	853	202	0	20	2330	6.3	43.2
	247	353	647	524	496	0	20	2330	6.3	39.7
	271	353	625	0	959	0	20	2330	6.3	43.3
	206	353	661	864	216	0	20	2330	6.3	43

	207	353	649	531	531	0	20	2330	6.3	38.1
[4]	165	300	765	905	267	4.98	25	2430	4.4	42
	165	318	739	608	537	6.042	25	2430	4.4	41
	162	325	683	0	1123	6.175	25	2430	4.4	40
[5]	160.6	380	598	1182	52	4.9	20	2165	6.8	62.2
	165.4	380	529	1175	103	4.9	20	2165	6.8	58.4
	170.2	380	460	1168	154	4.9	20	2165	6.8	61.3
	175.6	380	327	1162	254	4.9	20	2165	6.8	60.8
	180.9	380	0	1162	509	4.9	20	2165	6.8	61
[6]	225	410	642	840	204	0	20	2570	3.5	45.3
	225	410	642	524	506	0	20	2570	3.5	42.5
	225	410	642	210	814	0	20	2570	3.5	39.2
	225	410	642	0	1017	0	20	2570	3.5	37.1
[7]	180	400	708	886	215	0	20	2570	3.5	62.4
	180	400	708	554	538	0	20	2570	3.5	55.8
	180	400	708	0	1075	0	20	2570	3.5	42
	225	410	642	840	204	0	20	2570	3.5	45.3
	225	410	642	524	506	0	20	2570	3.5	42.5
	225	410	642	0	1017	0	20	2570	3.5	38.1
[8]	234	360	705	0	1100	0	19	2390	4.4	22.1
	190	380	705	0	1100	0	19	2390	4.4	25.1
	192	400	705	0	1100	0	19	2390	4.4	27.2
	181	420	705	0	1100	0	19	2390	4.4	28.7
	184	460	705	0	1100	0	19	2390	4.4	29.5
[9]	178	264	835	0	1030	0	30	2520	3.8	18

	174	262	830	0	1020	0	30	2510	3.9	15.4
	148	427	760	0	1000	4.2	30	2520	3.8	36.4
	153	423	755	0	990	4.1	30	2510	3.9	35.7
	152	443	855	0	885	3.9	30	2520	3.8	44.4
[10]	225	410	642	840	204	0	20	2580	3.5	45.3
	225	410	642	524	506	0	20	2580	3.5	42.5
	225	410	642	0	1017	0	20	2580	3.5	38.1
	205	410	662	865	210	0	20	2580	3.5	51.7
	205	410	662	541	525	0	20	2580	3.5	47.1
	205	410	662	0	1049	0	20	2580	3.5	43.4
	180	400	708	886	215	5.6	20	2580	3.5	62.4
	180	400	708	554	538	5.6	20	2580	3.5	56.8
	180	400	708	0	1075	5.6	20	2580	3.5	52.1
	160	400	729	912	221	7.8	20	2580	3.5	69.6
	160	400	729	570	554	7.8	20	2580	3.5	65.3
	160	400	729	0	1107	7.8	20	2580	3.5	58.5
[11]	175	350	730	711	297	1.68	25	2530	1.9	36.7
	175	350	730	508	494	1.68	25	2530	1.9	38
	175	350	730	0	989	1.68	25	2530	1.9	36
	175	350	730	508	469	1.68	25	2400	6.2	30.4
	175	350	730	0	938	1.68	25	2400	6.2	29.5
[12]	190	380	744.45	756.97	189.24	2.66	20	2338	5.2	47.4
	190	380	709.54	471.13	471.12	2.66	20	2338	5.2	47.3
	190	380	714.56	0	874.04	5.32	20	2338	5.2	54.8
	140	350	732	519	556	4.2	12	2420	6.8	43.3

[13]	153	340	723	512	549	3.4	12	2400	6.8	39.6
	165	330	715	507	543	2.64	12	2400	6.8	38.1
	176	320	708	502	537	1.92	12	2400	6.8	34.5
	186	310	702	497	533	1.24	12	2400	6.8	31.6
	140	350	732	553	523	4.2	22	2420	8.8	46.1
	153	340	723	547	517	3.4	22	2420	8.8	45.8
	165	330	715	541	511	2.64	22	2420	8.8	39.9
	176	320	708	535	506	1.92	22	2420	8.8	36.3
	186	310	702	531	501	1.24	22	2420	8.8	34.7
[14]	200	270	750	675	200	1.08	19	2440	5.8	18.5
	210	270	750	450	400	1.35	19	2440	5.8	18.0
	220	270	750	225	600	1.62	19	2440	5.8	16.5
	165	370	865	760	230	1.48	19	2440	5.8	33.0
	165	370	865	505	455	1.85	19	2440	5.8	34.5
	165	370	865	250	680	2.59	19	2440	5.8	34.0
[15]	178.5	275	938.05	723.07	180.77	1.925	16	2400	5	31.7
	178.5	275	962.73	423.77	423.77	1.925	16	2400	5	32.4
	178.5	275	1005.18	0	756.46	1.925	16	2400	5	30.1
	190	380	794.31	750.04	187.57	2.66	16	2400	5	43.7
	190	380	811.37	443.71	443.71	2.66	16	2400	5	37.5
	190	380	838.29	0	807.97	2.66	16	2400	5	40.5
[15]	151	335	630	414	720	1.266	19	2420	5.4	41.4
	156	349	888	0	792	1.67616	19	2420	5.4	43.9
	161	358	645	281	813	1.3584	19	2500	3.3	44.8
	156	349	857	0	867	1.2564	19	2500	3.3	45.9

[16]	172.43	401	574	911	303	0.2005	20	2661	1.9	47
	172.43	401	574	585	585	0.70175	20	2602	2.6	46
	172.43	401	574	0	1119	0.90225	20	2510	3.9	42.5
[17]	190.8	424	770	0	980	0	19	2490	4.8	41
	192.5	350	800	0	1015	0	19	2490	4.8	33.3
	191.75	295	814	0	1039	0	19	2490	4.8	24.8
[18]	175	325	0	0	1762	3.45	32	2263	6	33.2
	222	350	0	0	1778	4.5	32	2283	4.2	35.6
	221	350	0	0	1771	4.5	32	2292	4.3	34.6
	195	325	0	0	1710	3.25	32	2301	5	37.3
	123	300	0	192	1728	3	32	2609	1.5	45.4
	144	325	0	768	1152	3.25	32	2518	2.7	54.3
	123	325	0	754.4	1131.6	3.25	32	2584	1.6	54.4
	132	300	0	1448.25	482.75	3	32	2594	1.6	53.4
[19]	180	275	625	882	378	0	20	2340	5.3	20.0
	180	295	595	635	635	0	20	2340	5.3	19.0
	180	310	610	0	1240	0	20	2340	5.3	18.0
	180	330	585	872	373	0	20	2340	5.3	23.0
	180	355	560	623	623	0	20	2340	5.3	24.0
	180	372	536	0	1252	0	20	2340	5.3	21
	180	355	560	872	373	0	20	2340	5.3	25.0
	180	385	550	613	613	0	20	2340	5.3	29.0
	180	409	525	0	1226	0	20	2340	5.3	30
	180	375	544	869	372	0	20	2340	5.3	39

	180	405	508	624	624	0	20	2340	5.3	31.0
	180	426	494	0	1241	0	20	2340	5.3	34
[20]	193	350	661	1061	57	0	12	2010	10.9	40
	194	350	515	1061	170	0	12	2010	10.9	38.6
	196	350	368	1061	283	0	12	2010	10.9	37.6
	199	158	0	1061	566	0	12	2010	10.9	38.6
	158	350	693	1111	59	3.5	12	2010	10.9	53.7
	163	350	536	1105	177	3.5	12	2010	10.9	51
	168	350	381	1100	294	3.5	12	2010	10.9	47.8
	178	350	0	1089	582	3.5	12	2010	10.9	45.1
	137	350	713	1143	61	3.5	12	2010	10.9	64.6
	139	350	555	1143	183	3.5	12	2010	10.9	65.4
	143	350	395	1138	304	3.5	12	2010	10.9	63.2
	150	350	0	1132	605	3.5	12	2010	10.9	63.0
[21]	180	281	802	0	970	0	10	2360	4.7	38.6
	170	293	648	0	919	0	10	2280	6.2	38.1
	165	337	841	0	879	0	10	2220	7.8	39.3
	190	463	621	0	970	0	10	2360	4.7	60.1
	190	500	621	0	919	3.24	10	2280	6.2	60.2
	180	600	567	0	879	5.04	10	2220	7.8	62.8
	220	537	693	782	138	0	20	2330	4.4	50.8
[22]	220	537	693	644	276	0	20	2330	4.4	44.9
	220	537	693	506	414	0	20	2330	4.4	44.6
	220	537	693	368	552	0	20	2330	4.4	42.4
	220	537	693	782	138	0	20	2370	4	54

	220	537	693	644	276	0	20	2370	4	56
	220	537	693	506	414	0	20	2370	4	54.4
	220	537	693	368	552	0	20	2370	4	40.6
	220	537	693	782	138	0	20	2390	3.6	55.2
	220	537	693	644	276	0	20	2390	3.6	53.5
	220	537	693	506	414	0	20	2390	3.6	56.9
	220	537	693	368	552	0	20	2390	3.6	54.7
	220	537	693	782	138	0	20	2320	4.6	50.5
	220	537	693	644	276	0	20	2320	4.6	48.9
	220	537	693	506	414	0	20	2320	4.6	45.8
	220	537	693	368	552	0	20	2320	4.6	40
	220	537	693	782	138	0	20	2390	3.7	54.4
	220	537	693	644	276	0	20	2390	3.7	50.2
	220	537	693	506	414	0	20	2390	3.7	49.5
	220	537	693	368	552	0	20	2390	3.7	40.4
	220	537	693	782	138	0	20	2390	3.5	45
	220	537	693	644	276	0	20	2390	3.5	46.9
	220	537	693	506	414	0	20	2390	3.5	51.4
	220	537	693	368	552	0	20	2390	3.5	53.2
	220	537	693	782	138	0	20	2380	3.8	55.3
	220	537	693	644	276	0	20	2380	3.8	55.9
	220	537	693	506	414	0	20	2380	3.8	52.6
	220	537	693	368	552	0	20	2380	3.8	48
	220	537	693	782	138	0	20	2380	3.8	49.1

	220	537	693	644	276	0	20	2380	3.8	49.9
	220	537	693	506	414	0	20	2380	3.8	50.3
	220	537	693	368	552	0	20	2380	3.8	47.5
	220	537	693	782	138	0	20	2400	3.5	43.2
	220	537	693	644	276	0	20	2400	3.5	53.7
	220	537	693	506	414	0	20	2400	3.5	50
	220	537	693	368	552	0	20	2400	3.5	43.3
	220	537	693	782	138	0	20	2370	4	52.9
	220	537	693	644	276	0	20	2370	4	49.9
	220	537	693	506	414	0	20	2370	4	53.7
	220	537	693	368	552	0	20	2370	4	46
[23]	206	413	606	0	987	0	25	2452	4.1	51
	206	413	606	0	987	0	25	2452	4.1	49
	206	413	606	0	987	0	25	2452	4.1	48
	206	413	606	537	494	0	25	2452	4.1	51
	206	413	606	537	494	0	25	2452	4.1	51
	206	413	606	537	494	0	25	2452	4.1	51
	206	413	606	805	245	0	25	2452	4.1	52
	206	413	606	805	245	0	25	2452	4.1	50
	206	413	606	805	245	0	25	2452	4.1	49
[24]	145.6	520	577.2	0	1040	0	25	2260	7.5	38.3
	145.6	520	577.2	0	1040	0	25	2260	7.5	32.9
	119.6	520	577.2	0	1040	0	25	2260	7.5	33.2
	146.2	430	653.6	0	1032	0	25	2260	7.5	31.3

	146.2	430	653.6	0	1032	0	25	2260	7.5	28.4
	120.4	430	653.6	0	1032	0	25	2260	7.5	28.0
	145.77	339	728.85	0	1050.9	0	25	2260	7.5	26.5
	145.77	339	728.85	0	1050.9	0	25	2260	7.5	23.3
	118.65	339	728.85	0	1050.9	0	25	2260	7.5	21.6
	144.06	294	767.34	0	1029	0	25	2260	7.5	21.6
	144.06	294	767.34	0	1029	0	25	2260	7.5	18.0
	117.6	294	767.34	0	1029	0	25	2260	7.5	18.8
	146.91	249	804.27	0	1045.8	0	25	2260	7.5	16.1
	146.91	249	804.27	0	1045.8	0	25	2260	7.5	13.4
	119.52	249	804.27	0	1045.8	0	25	2260	7.5	13.9
[25]	179	275	878	735	184	0	20	2320	5.3	41
	179	275	849	455	455	0	20	2320	5.3	44
	179	275	868	0	830	0	20	2320	5.3	45
	190	380	744	757	189	0	20	2320	5.3	50.5
	190	380	710	471	471	0	20	2320	5.3	45
	190	380	715	0	874	0	20	2320	5.3	56
	179	275	961	740	185	0	20	2320	5.3	33.5
	179	275	978	408	408	0	20	2320	5.3	32
	179	275	1010	0	640	0	20	2320	5.3	32
	190	380	813	767	192	0	20	2320	5.3	44
	190	380	822	426	427	0	20	2320	5.3	41
	190	380	836	0	683	0	20	2320	5.3	41.5
	179	325	799	839	210	0	20	2320	5.3	44
	179	325	831	490	490	0	20	2320	5.3	41

	179	325	825	0	923	0	20	2320	5.3	33.5
	173	385	698	892	223	0	20	2320	5.3	53.5
	173	385	742	515	515	0	20	2320	5.3	54
	173	385	746	0	963	0	20	2320	5.3	40
[26]	159.6	380	862.4	489.3	489.3	5.7	20	2330	6.1	41.6
	193.8	380	934.1	0	867.7	6.46	20	2330	6.1	31.4
	197.6	380	862.4	489.3	489.3	5.7	20	2330	6.1	35.5
	231.8	380	934.1	0	867.7	6.46	20	2330	6.1	26
	167.2	380	862.4	489.3	489.3	5.7	20	2320	5.8	44.6
	193.8	380	934.1	0	867.7	6.46	20	2320	5.8	36.7
	235.6	380	934.1	0	867.7	6.46	20	2320	5.8	29.5
	155.8	380	818.5	840.9	210.2	4.56	20	2360	3.9	46.1
	159.6	380	862.4	489.3	489.3	5.7	20	2360	3.9	45.1
	171	380	934.1	0	867.7	6.46	20	2360	3.9	42.9
	190	380	818.5	840.9	210.2	4.56	20	2360	3.9	39.3
	197.6	380	862.4	489.3	489.3	5.7	20	2360	3.9	39.5
	205.2	380	934.1	0	867.7	6.46	20	2360	3.9	37.7
	159.6	380	818.5	840.9	210.2	4.56	20	2350	4.5	48.1
	163.4	380	862.4	489.3	489.3	5.7	20	2350	4.5	41
	152	380	934.1	0	867.7	6.46	20	2350	4.5	38.7
	193.8	380	818.5	840.9	210.2	4.56	20	2350	4.5	42.7
	197.6	380	862.4	489.3	489.3	5.7	20	2350	4.5	35.4
	190	380	934.1	0	867.7	6.46	20	2350	4.5	31.4
	159.6	380	818.5	840.9	210.2	4.56	20	2350	4.7	48.5

	159.6	380	862.4	489.3	489.3	5.7	20	2350	4.7	45.4
	163.4	380	934.1	0	867.7	6.46	20	2350	4.7	37
	197.6	380	818.5	840.9	210.2	4.56	20	2350	4.7	41.3
	197.6	380	862.4	489.3	489.3	5.7	20	2350	4.7	36.8
	212.8	380	934.1	0	867.7	6.46	20	2350	4.7	31.2
[27]	159.8	340	556	1020	238	0	20	2336	3.6	50
	159.8	340	556	638	596	0	20	2315	3.6	45.3
	159.8	340	556	319	894	0	20	2295	3.6	44
[28]	185.4	309	864	848	211	1.0197	16	2380	6.9	42.9
	191.7	320	817.5	538	538	1.056	16	2380	6.9	42.5
	201.6	336	785	0	1060	1.1088	16	2380	6.9	40.9
	192.5	386	829	808	202	2.0458	16	2380	6.9	51.6
	200	399	795	504	504	2.1147	16	2380	6.9	51.6
	210	420	738	0	1014	2.226	16	2380	6.9	50.3
[29]	205	300	697	0	1075	0	20	2450	3.1	35
	205	300	697	0	1027	0	20	2370	7.1	29.2
	205	300	697	0	1027	0	20	2360	7.8	27.7
	180	350	706	0	1089	0	20	2450	3.1	47.6
	180	350	706	0	1041	0	20	2370	7.1	42
	180	350	706	0	1041	0	20	2360	7.8	42.9
	185	425	696	0	1028	0	20	2450	3.1	60
	185	425	696	0	982	0	20	2370	7.1	53.7
	185	425	696	0	982	0	20	2360	7.8	53.2
	165	485	685	0	1039	0	20	2450	3.1	78.2
	165	485	685	0	979	0	20	2370	7.1	71.2

	165	485	685	0	982	0	20	2360	7.8	65.4
[30]	178.3	358	730.4	783.6	299.3	0.3	19	2570	2.7	33.6
	178.3	358	730.4	458.3	598.4	0.3	19	2570	2.7	30.4
	178.3	358	730.4	0	1020	0.3	19	2570	2.7	29.1
[31]	195	300	787.1	756.4	189.1	0	20	2300	5.2	39.5
	195	300	737.4	485.5	485.5	0	20	2300	5.2	40.8
	195	300	712.6	0	951.4	0	20	2300	5.2	43.7
	195	300	814.4	733	183.2	0	20	2300	5.5	41
	195	300	804.2	450.7	450.7	0	20	2300	5.5	38.8
	195	300	807.9	0	855.2	0	20	2300	5.5	39.9
[32]	214.2	210	929	0	966	0	22	2451	7.8	19.7
	196	280	866	0	940	0	22	2387	6.9	35.7
	161	350	858	0	974	3.5	22	2362	4.2	66.8
	212.1	210	932	0	970	0	22	2456	7.5	21.8
	193.2	280	870	0	970	0	22	2455	6.4	36.1
	157.5	350	858	0	1029	3.5	22	2496	4.2	68.5
	207.9	210	938	0	953	0	22	2401	7.6	21
	187.6	280	877	0	988	0	22	2484	5.4	41.1
	150.5	350	868	0	982	3.5	22	2363	3.6	70.2
	205.8	210	943	0	977	0	22	2447	6.9	23.6
	190.4	280	873	0	962	0	22	2458	5.8	39.7
	157.5	350	858	0	1016	3.5	22	2464	3.9	66.5
[33]	179	275	878	735	184	0	19	2320	5.3	49.3
	179	275	849	455	455	0	19	2320	5.3	47.5
	179	275	868	0	830	0	19	2320	5.3	53.7

	190	380	714	757	189	0	19	2320	5.3	64.8
	190	380	710	471	471	0	19	2320	5.3	63.5
	190	380	715	0	874	0	19	2320	5.3	65.1
	179	275	961	740	185	0	19	2320	5.3	64.8
	179	275	978	408	408	0	19	2320	5.3	63.5
	179	275	1010	0	640	0	19	2320	5.3	65.1
	190	380	813	767	192	0	19	2320	5.3	54.9
	190	380	822	426	427	0	19	2320	5.3	51.5
	190	380	836	0	683	0	19	2320	5.3	50.3
	179	325	799	839	210	0	19	2320	5.3	56.5
	179	325	831	490	490	0	19	2320	5.3	48.9
	179	325	825	0	923	0	19	2320	5.3	43.1
	173	385	698	892	233	0	19	2320	5.3	67.4
	173	385	742	515	515	0	19	2320	5.3	61.2
	173	385	746	0	963	0	19	2320	5.3	53.7

References

1. Limbachiya, M.C.; Leelawat, T.; Dhir, R.K. Use of recycled concrete aggregate in high-strength concrete. *Mater. Struct.* **2000**, *33*, 574–580, https://doi.org/10.1007/bf02480538.
2. Gómez-Soberón, J.M.V. Porosity of recycled concrete with substitution of recycled concrete aggregate: An experimental study. *Cem. Concr. Res.* **2002**, *32*, 1301–1311, https://doi.org/10.1016/s0008-8846(02)00795-0.
3. Poon, C.S.; Shui, Z.H.; Lam, L.; Fok, H.; Kou, S.C. Influence of moisture states of natural and recycled aggregates on the slump and compressive strength of concrete. *Cem. Concr. Res.* **2004**, *34*, 31–36, https://doi.org/10.1016/s0008-8846(03)00186-8.
4. Etxeberria, M.; Mari, A.; Vázquez, E. Recycled aggregate concrete as structural material. *Mater. Struct.* **2006**, *40*, 529–541, https://doi.org/10.1617/s11527-006-9161-5.

5. Evangelista, L.; de Brito, J. Mechanical behaviour of concrete made with fine recycled concrete aggregates. *Cem. Concr. Compos.* **2007**, *29*, 397–401, https://doi.org/10.1016/j.cemconcomp.2006.12.004.
6. Poon, C.S.; Kou, S.C.; Lam, L. Influence of recycled aggregate on slump and bleeding of fresh concrete. *Mater. Struct.* **2006**, *40*, 981–988, https://doi.org/10.1617/s11527-006-9192-y.
7. Kou, S.C.; Poon, C.S.; Chan, D. Influence of Fly Ash as Cement Replacement on the Properties of Recycled Aggregate Concrete. *J. Mater. Civ. Eng.* **2007**, *19*, 709–717, https://doi.org/10.1061/(asce)0899-1561(2007)19:9(709).
8. Rahal, K. Mechanical properties of concrete with recycled coarse aggregate. *Build. Environ.* **2007**, *42*, 407–415, https://doi.org/10.1016/j.buildenv.2005.07.033.
9. Casuccio, M.; Torrijos, M.; Giaccio, G.; Zerbino, R. Failure mechanism of recycled aggregate concrete. *Constr. Build. Mater.* **2008**, *22*, 1500–1506, https://doi.org/10.1016/j.conbuildmat.2007.03.032.
10. Kou, S.C.; Poon, C.S.; Chan, D. Influence of fly ash as a cement addition on the hardened properties of recycled aggregate concrete. *Mater. Struct.* **2007**, *41*, 1191–1201, https://doi.org/10.1617/s11527-007-9317-y.
11. Yang, K.-H.; Chung, H.-S.; Ashour, A.F. Influence of Type and Replacement Level of Recycled Aggregates on Concrete Proper-ties. *ACI Mater. J.* **2008**, *105*, 289–296
12. Domingo-Cabo, A.; Lázaro, C.; López-Gayarre, F.; Serrano-López, M.A.; Serna, P.; Castaño-Tabares, J.O. Creep and shrinkage of recycled aggregate concrete. *Constr. Build. Mater.* **2009**, *23*, 2545–2553, doi:10.1016/j.conbuildmat.2009.02.018.
13. Corinaldesi, V. Mechanical and elastic behaviour of concretes made of recycled-concrete coarse aggregates. *Constr. Build. Mater.* **2010**, *24*, 1616–1620, https://doi.org/10.1016/j.conbuildmat.2010.02.031.
14. Zega, C.J.; Di Maio, .A. Recycled Concretes Made with Waste Ready-Mix Concrete as Coarse Aggregate. *J. Mater. Civ. Eng.* **2011**, *23*, 281–286, https://doi.org/10.1061/(asce)mt.1943-5533.0000165.
15. Fathifazl, G.; Razaqpur, A.G.; Isgor, O.B.; Abbas, A.; Fournier, B.; Foo, S. Creep and drying shrinkage characteristics of concrete produced with coarse recycled concrete aggregate. *Cem. Concr. Compos.* **2011**, *33*, 1026–1037, https://doi.org/10.1016/j.cemconcomp.2011.08.004.
16. Rao, M.C.; Bhattacharyya, S.K.; Barai, S.V. Influence of field recycled coarse aggregate on properties of concrete. *Mater. Struct.* **2010**, *44*, 205–220, https://doi.org/10.1617/s11527-010-9620-x.
17. Somna, R.; Jaturapitakkul, C.; Chalee, W.; Rattanachu, P. Effect of the Water to Binder Ratio and Ground Fly Ash on Properties of Recycled Aggregate Concrete. *J. Mater. Civ. Eng.* **2012**, *24*, 16–22, https://doi.org/10.1061/(asce)mt.1943-5533.0000360.
18. Hoffmann, C.; Schubert, S.; Leemann, A.; Motavalli, M. Recycled concrete and mixed rubble as aggregates: Influence of variations in composition on the concrete properties and their use as structural material. *Constr. Build. Mater.* **2012**, *35*, 701–709, https://doi.org/10.1016/j.conbuildmat.2011.10.007.
19. Limbachiya, M.; Meddah, M.S.; Ouchagour, Y. Performance of Portland/Silica Fume Cement Concrete Produced with Recycled Concrete Aggregate. *ACI Mater. J.* **2012**, *109*, 91–.
20. Pereira, P.; Evangelista, L.; de Brito, J. The effect of superplasticizers on the mechanical performance of concrete made with fine recycled concrete aggregates. *Cem. Concr. Compos.* **2012**, *34*, 1044–1052, https://doi.org/10.1016/j.cemconcomp.2012.06.009.
21. Butler, L.; West, J.S.; Tighe, S.L. Effect of recycled concrete coarse aggregate from multiple sources on the hardened properties of concrete with equivalent compressive strength. *Constr. Build. Mater.* **2013**, *47*, 1292–1301, https://doi.org/10.1016/j.conbuildmat.2013.05.074.
22. Ismail, S.; Ramli, M. Engineering properties of treated recycled concrete aggregate (RCA) for structural applications. *Constr. Build. Mater.* **2013**, *44*, 464–476, https://doi.org/10.1016/j.conbuildmat.2013.03.014.
23. Matias, D.; de Brito, J.; Rosa, A.; Pedro, D. Mechanical properties of concrete produced with recycled coarse aggregates – Influence of the use of superplasticizers. *Constr. Build. Mater.* **2013**, *44*, 101–109, https://doi.org/10.1016/j.conbuildmat.2013.03.011.

24. Sheen, Y.-N.; Wang, H.-Y.; Juang, Y.-P.; Le, D.-H. Assessment on the engineering properties of ready-mixed concrete using recycled aggregates. *Constr. Build. Mater.* **2013**, *45*, 298–305, https://doi.org/10.1016/j.conbuildmat.2013.03.072.
25. Thomas, C.; Setién, J.; Polanco, J.; Alaejos, P.; de Juan, M.S. Durability of recycled aggregate concrete. *Constr. Build. Mater.* **2013**, *40*, 1054–1065, https://doi.org/10.1016/j.conbuildmat.2012.11.106.
26. Ulloa, V.A.; García-Taengua, E.; Pelufo, M.-J.; Domingo, A.; Serna, P. New views on effect of recycled aggregates on concrete compressive strength. *ACI Mater. J.* **2013**, *110*, 1–10.
27. Younis, K.H.; Pilakoutas, K. Strength prediction model and methods for improving recycled aggregate concrete. *Constr. Build. Mater.* **2013**, *49*, 688–701.
28. Beltrán, M.G.; Barbudo, A.; Agrela, F.; Galvín, A.P.; Jiménez, J.R. Effect of cement addition on the properties of recycled concretes to reach control concretes strengths. *J. Clean. Prod.* **2014**, *79*, 124–133, https://doi.org/10.1016/j.jclepro.2014.05.053.
29. Duan, Z.; Poon, C.S. Properties of recycled aggregate concrete made with recycled aggregates with different amounts of old adhered mortars. *Mater. Des.* **2014**, *58*, 19–29, https://doi.org/10.1016/j.matdes.2014.01.044.
30. Folino, P.; Xargay, H. Recycled aggregate concrete – Mechanical behavior under uniaxial and triaxial compression. *Constr. Build. Mater.* **2014**, *56*, 21–31, https://doi.org/10.1016/j.conbuildmat.2014.01.073.
31. Gayarre, F.L.; Pérez, C.L.-C.; López, M.A.S.; Cabo, A.D. The effect of curing conditions on the compressive strength of recycled aggregate concrete. *Constr. Build. Mater.* **2014**, *53*, 260–266, https://doi.org/10.1016/j.conbuildmat.2013.11.112.
32. Pedro, D.; de Brito, J.; Evangelista, L. Performance of concrete made with aggregates recycled from precasting industry waste: influence of the crushing process. *Mater. Struct.* **2014**, *48*, 3965–3978, https://doi.org/10.1617/s11527-014-0456-7.
33. Thomas, C.; Sosa, I.; Setién, J.; Polanco, J.A.; Cimentada, A.I. Evaluation of the fatigue behavior of recycled aggregate concrete. *J. Clean. Prod.* **2014**, *65*, 397–405, https://doi.org/10.1016/j.jclepro.2013.09.036.
